# Establishment and Verification of a Predictive Nomogram for New Vertebral Compression Fracture Occurring after Bone Cement Injection in Middle‐Aged and Elderly Patients with Vertebral Compression Fracture

**DOI:** 10.1111/os.13655

**Published:** 2023-01-31

**Authors:** Wenxin Gao, Yungang Chen, Xiaoying Wang, Guoyan Liu, Kaiying Cui, Jinxing Guo, Jianhu Zheng, Yanke Hao

**Affiliations:** ^1^ Shandong University of Traditional Chinese Medicine Jinan China; ^2^ Jinan Vocational College of Nursings Jinan China; ^3^ Shandong University of Traditional Chinese Medicine Affiliated Hospital Jinan China

**Keywords:** Compression Fractures, New Vertebral Compression Fractures, Nomogram, Percutaneous Kyphoplasty, Percutaneous Vertebroplasty, Risk Factors

## Abstract

**Objective:**

New vertebral compression fracture (NVCF) occurring after bone cement injection in middle‐aged and elderly patients with vertebral compression fracture is very common. Preoperative baseline characteristics and surgical treatment parameters have been widely studied as a risk factor, but the importance of the patients' laboratory indicators has not been thoroughly explored. We aimed to explore the relationship between laboratory indicators and NVCF, and attempt to construct a clinical prediction model of NVCF together with other risk factors.

**Methods:**

Retrospective analysis was performed for 200 patients who underwent bone cement injection (percutaneous kyphoplasty or vertebroplasty) for vertebral compression fractures between January 2019 and January 2020. We consulted the relevant literature and collated the factors affecting the occurrence of NVCF. Feature selection of patients with NVCF was optimized using the least absolute shrinkage and selection operator regression model, which was used to conduct multivariable logistic regression analysis, to create a predictive model incorporating the selected features. The discrimination, calibration, and clinical feasibility of the predictive model were assessed using the concordance index (C‐index), calibration plots, and decision curve analysis. Internal validation was performed using Bootstrap resampling verification.

**Results:**

Time from injury to surgery exceeding 7 days, low osteocalcin levels, elevated homocysteine levels, osteoporosis, mode of operation (percutaneous vertebroplasty), lack of postoperative anti‐osteoporosis treatment, and poor diffusion of bone cement were independent risk factors for NVCF in middle‐aged and elderly patients with vertebral compression fracture after bone cement injection. The C‐index of the nomogram constructed using these seven factors was 0.895, indicating good discriminatory ability. The calibration plot showed that the model was well calibrated. Bootstrap resampling verification yielded a significant C‐index of 0.866. Decision curve analysis demonstrated that the greatest clinical net benefit for predicting NVCF after bone cement injection could be achieved with a threshold of 1%–91%.

**Conclusion:**

This nomogram can effectively predict NVCF incidence after bone cement injection in middle‐aged and elderly patients with vertebral compression fracture, thus aiding clinical decision‐making and postoperative management, promoting effective postoperative rehabilitation, and improving the quality of life.

## Graphical abstract

Our study is the first study to combine patients' laboratory indicators, especially osteocalcin and homocysteine, with preoperative baseline characteristics and surgical treatment parameters into an innovative, easy‐to‐use nomogram for risk prediction, and to evaluate the risk of NVCF in middle‐aged and elderly patients with vertebral compression fractures after cement injection.

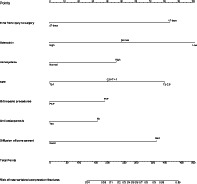

## Introduction

Vertebral compression fractures caused by minor trauma or no obvious trigger are the most common fragility fractures.^1^ Pain and limitation of movement caused by fracture are the principal factors affecting the quality of life, and may even result in disability in middle‐aged and elderly patients.^2^ Due to the aging of the population, vertebral compression fracture has led to myriad medical and healthcare problems.^1^ Currently, percutaneous kyphoplasty (PKP) and percutaneous vertebroplasty (PVP) are the most frequently used and successful minimally invasive surgical procedures for vertebral compression fractures, which can effectively ameliorate pain and facilitate rapid recovery.^3^, ^4^, ^5^ However, case reports and series describing postoperative new vertebral compression fracture (NVCF) have garnered considerable attention, owing to the popularity of PKP and PVP. According to studies, the prevalence of NVCF is approximately 2%–23% after PKP and 2.4%–52% after PVP.^6^ Meanwhile, NVCF, as one of the most serious complications after bone cement injection, may lead to more severe local pain, which brings a huge psychological and economic burden to patients. Therefore, more clinicians and scholars have begun to pay attention to it and explore the occurrence and development of NVCF.^6^ However, the cause of NVCF remains unclear. The known risk factors for NVCF include preoperative demographic characteristics such as osteoporosis and advanced patient age, and surgical factors such as bone cement leakage and lack of anti‐osteoporosis treatment.^7^, ^8^ Nevertheless, laboratory indicators such as osteocalcin and homocysteine that can reflect the condition of bone metabolism have not been studied in detail.

Therefore, this study aimed to investigate laboratory indicators, preoperative baseline characteristics, and surgical treatment‐related parameters as possible risk factors, to explore the relationship between them and the occurrence of NVCF, and to construct a predictive nomogram. A nomogram is a predictive model that estimates the risk of a specific disease by integrating several factors for complex operations.^9^ It can aid in comprehensive evaluation of the probability of postoperative NVCF, and implementation of targeted and personalized preoperative and postoperative management for high‐risk patients, with the objective of reducing the incidence of postoperative NVCF.

## Methods

### Patient Population

This study entailed a retrospective analysis of 200 patients who underwent bone cement injection (PKP or PVP) for vertebral compression fractures at the Affiliated Hospital of Shandong University of Traditional Chinese Medicine between January 2019 and January 2020. All patients were monitored for 2 years. The study protocol was approved by the Affiliated Hospital of Shandong University of Traditional Chinese Medicine's institutional research ethics committee. All patients received an explanation of the PVP or PKP procedure and clinical data processing. We also obtained written informed consent from all patients. This investigation complied with the requirements of Transparent Reporting of a Multivariable Prediction Model for Individual Prognosis or Diagnosis.^10^

Patients who meet the following criteria were included in our study: (1) complete preoperative basic information, and laboratory and imaging examination of the patients were available, which were re‐examined at the prescribed time postoperatively; (2) patients treated with PVP or PKP; (3) patients who presented with severe back pain and limited physical activity, especially when turning over or getting up; (4) patients with a fresh fracture confirmed by magnetic resonance imaging (MRI), who were diagnosed with a single vertebral compression fracture using X‐ray and computed tomography; (5) the fracture did not involve the posterior wall of the vertebral body, which was intact; (6) patients with spontaneous fractures or fractures caused by minor trauma; (7) patients who did not develop any infection within 15 cm of the puncture site; (8) patients without cardiopulmonary, liver, and renal failure; and (9) patients without coagulopathy or bleeding tendency. The exclusion criteria for the study were as follows: (1) patients with vertebral compression fracture involving more than two segments; (2) patients with pathological vertebral compression fracture caused by vertebral malignant tumor, metastatic tumor, haemangioma, and so forth; (3) patients with unstable fracture and burst fracture of the posterior wall of the vertebral body; (4) patients with severe spinal degeneration; (5) patients with a history of malignant tumors, dementia, or other mental disorders; (6) patients with insufficient clinical or imaging data; (7) patients with spinal cord compression and obvious neurological symptoms. such as numbness and/or muscle weakness; (8) patients with blood coagulation dysfunction, complications associated with systemic disease, and unable to tolerate surgery; and (9) patients with systemic or local infection. Key researchers extracted the data from the electronic medical records, and image archiving and communication systems.

### Percutaneous Kyphoplasty and Vertebroplasty

An experienced chief physician performed all procedures in this study using a unilateral lateral approach to the pedicle of the vertebral arch. The size of the vertebral body and level of compression and vertebral body leakage were used to calculate the dose of bone cement to be injected. The patient was placed in the prone position, with supportive padding to allow the abdomen to hang freely. We used C‐arm fluoroscopy to determine and mark the needle entry point, which was located at the lateral side of the pedicle of the vertebral arch on the right side of the vertebral body. After successful local anesthesia, a cement needle was used to puncture the lateral pedicle of the vertebral arch on the right side of the middle and posterior third of the vertebral body under fluoroscopy. The tip of the cement needle puncture was placed 3–4 cm from the spine at an abducent angle of 30°–45°. Thereafter, the needle core was withdrawn, and the solid cone drill was rotated through the puncture channel to the needle entry site under fluoroscopy. This site was located at the anterior and middle third of the vertebral body under lateral fluoroscopy, and at the spinous process of the vertebral body under anterior fluoroscopy. The solid vertebral drill was withdrawn, followed by the insertion of a balloon catheter for vertebral dilatation to facilitate gradual pressurization and maintenance of the balloon at a certain pressure. The height of the vertebral body was restored partially, and the balloon was pulled out under fluoroscopy. The bone cement was mixed to toothpaste consistency, and slowly injected into the vertebral body via the bone cement filler under fluoroscopy. After the bone cement had hardened, we slowly removed the puncture needle to ensure that there was no leakage of bone cement. Finally, we covered the needle puncture site with sterile patches.

PVP did not entail insertion of a balloon catheter for vertebral dilatation, while the other surgical steps were the same as those for PKP.

### Identification Criteria for Outcome Measures

The principal diagnostic criteria of NVCF after PVP/PKP were as follows. (1) After initial pain amelioration, the patient experienced a resurgence of back pain within 2 years of surgery, which was accompanied by discernible local tenderness and limited physical activity, especially when turning over or getting up. (2) The vertebral body exhibited some wedge‐shaped changes on X‐ray, and MRI revealed low‐ and high‐signal intensities on the T1‐ and T2‐weighted sequences, respectively. These findings confirmed the existence of a NVCF. MRI was also utilized to eliminate the possibility of other spinal disorders, such as infections and malignant tumors.

### Selection Criteria for Predictor Variables

We conducted a comprehensive review of the previous literature, and collected and analyzed the postoperative risk factors associated with NVCF, such as laboratory indicators, baseline characteristics, and surgical treatment‐related factors. The patients' baseline characteristics included socio‐demographic factors [age, sex, time from injury to surgery, history of trauma, body mass index (BMI), bone mineral density (BMD)] and preoperative imaging examination indices (preoperative vertebral height, preoperative compression ratio, average vertebral height, segmental kyphosis angle, thoracolumbar kyphosis Cobb Angle, and coronal Cobb Angle). The surgical treatment factors included fracture site, orthopaedic procedures, and amount of bone cement injected. The laboratory indicators included parathyroid hormone, vitamin D, osteocalcin, β collagen special sequence, total type I collagen amino elongation peptide, red blood cell distribution width, fibrinogen, hemoglobin, activated partial thromboplastin time, homocysteine, alkaline phosphatase, serum Ca, and serum creatinine. Postoperative imaging changes (such as the diffusion of bone cement, vertebral height recovery rate, and whether the bone cement contacted the endplate) were also included in our study. We also investigated whether patients were administered postoperative anti‐osteoporosis therapy with zoledronic acid.

### Statistical Analysis

Statistical analysis, development, and verification of the model were conducted using SPSS 26.0 statistical software (International Business Machines Corporation, America), R software (The R Foundation for Statistical Computing, Austria, Version 4.2.0; https://www.R-project.org), and RStudio software (Public Benefit Corporation in Delaware, America, Version 2022.07.2–576; https://www.rstudio.com/). Continuous variables were presented as the mean ± SD, and count data were transformed into count classification variables, expressed as ratios. *p*‐values <0.05 were considered statistically significant.

The least absolute shrinkage and selection operator (LASSO) regression method restricts the coefficients of the predictive variables by imposing constraints on the process of parameter estimation, reduces the regression coefficients of independent predictive variables to zero, and minimizes the possibility of over‐fitting of the model.^11^ We screened the predictability of the ideal risk variables with non‐zero coefficients in the LASSO regression model using the LASSO approach. Thereafter, coupled with the characteristics selected in the LASSO regression model, the predictive model was established using multivariate logistic regression analysis. To assess the model's level of calibration, we created a calibration chart for the clinical predictive model. The area under the receiver operating characteristic curve indicates the accuracy with which a model can predict a future event. The net benefit under a tolerable risk threshold consistent with clinical practice was depicted using decision curve analysis, which was employed to assess the model's clinical feasibility. We applied Bootstrap validation resampling (1000 repetitions) to calculate the relative corrected concordance index. The statistical methods used in this study are supported by relevant literature^12^, ^13^ and supervised and reviewed by statistical experts at Shandong University of Traditional Chinese Medicine.

## Results

### Patients' Baseline Characteristics

A total of 200 patients satisfied the eligibility criteria for this study. Participants were divided into the non‐NVCF (*n* = 126) and NVCF groups (*n* = 74), based on the incidence of NVCF. All patients were monitored for 2 years and included in the final analysis. The laboratory indicators, baseline characteristics, and surgical treatment factors of the two groups are enumerated in Table 1.

**TABLE 1:** Baseline characteristics of patients with no new vertebral compression fractures and new vertebral compression fractures

Variables	Total (*n* = 200)	No new vertebral compression fractures (*n* = 126)	new vertebral compression fractures (*n* = 74)
Time from injury to surgery			
≤7 days	100(50)	86(68.3)	14(19)
>7 days	100(50)	40(31.7)	60(81)
History of trauma, *n* (%)			
No	52 (26)	25 (19.8)	27 (36.5)
Yes	148 (74)	101 (80.2)	47 (63.5)
Gender, *n* (%)			
Female	167 (83.5)	109 (86.5)	58 (78.4)
Male	33 (16.5)	17 (13.5)	16 (21.6)
Age(Mean ± SD)			
<65	24(12)	15(11.9)	9(12.1)
65 ≤ Age < 75	77(38.5)	50(39.7)	27(36.5)
75 ≤ Age < 85	75(37.5)	48(38.1)	27(36.5)
85≤	24(12)	13(10.3)	11(14.9)
BMI(Mean ± SD)			
<24	102(51)	59(46.8)	43(58.1)
24 < BMI ≤ 27.9	75(37.5)	52(41.3)	23(31.1)
>28	23(11.5)	15(11.9)	8(10.8)
Parathyroid Hormone			
Low	1(0.5)	1(0.8)	0(0)
Normal	172(86)	109(86.5)	63(85.1)
High	27(13.5)	16(12.7)	11(14.9)
Vitamin D			
Low	189(94.5)	120(95.2)	69(93.2)
Normal	11(5.5)	6(4.8)	5(6.8)
Osteocalcin			
Low	84(42)	45(35.7)	39(52.7)
Normal	112(56)	79(62.7)	33(44.6)
High	4(2)	2(1.6)	2(2.7)
β collagen special sequence			
Normal	88(44.0)	58(46.0)	30(40.5)
High	112(56.0)	68(54.0)	44(59.5)
Total type I collagen amino elongation peptide			
Normal	142(71)	107(84.9)	35(47.3)
High	58(29)	19(15.1)	39(52.7)
Red blood cell distribution width			
Low	3(1.5)	2(1.6)	1(1.4)
Normal	184(92)	113(89.7)	71(95.9)
High	13(6.5)	11(8.7)	2(2.7)
Fibrinogen			
Normal	81(40.5)	55(43.7)	26(35.1)
High	119(59.5)	71(56.3)	48(64.9)
Hematocrystallin			
Low	122(61)	77(61.1)	45(60.8)
Normal	78(39)	49(38.9)	29(39.2)
Activated partial thromboplastin time			
Low	4(2)	3(2.4)	1(1.4)
Normal	190(95)	117(92.8)	73(98.6)
High	6(3)	6(4.8)	0(0)
Homocysteine			
Normal	163(81.5)	107(84.9)	56(75.7)
High	37(18.5)	19(15.1)	18(24.3)
Alkaline phosphatase			
Low	1(0.5)	1(0.8)	(0)
Normal	143(71.5)	103(81.7)	40(54.1)
High	56(28)	22(17.5)	34(45.9)
Serum Ca			
Low	27(13.5)	16(12.7)	11(14.9)
Normal	173(86.5)	110(87.3)	63(85.1)
Serum creatinine			
Low	39(19.5)	22(17.5)	17(23.0)
Normal	151(75.5)	98(77.8)	53(71.6)
High	10(5)	6(4.7)	4(5.4)
BMD			
T ≥ ‐1.0	11(5.5)	9(7.1)	2(2.7)
−2.5 < T < −1.0	38(19)	30(23.9)	8(10.8)
T ≤ −2.5	151(75.5)	87(69.0)	64(86.5)
Amount of bone cement			
<4ml	56(28)	34(27.0)	22(29.7)
≥4ml, ≤ 6ml	117(58.5)	73(57.9)	44(59.5)
>6ml	27(13.5)	19(15.1)	8(10.8)
Orthopaedic procedures, *n* (%)			
PVP	71 (35.5)	38 (30.2)	33 (44.6)
PKP	129 (64.5)	88 (69.8)	41 (55.4)
Preoperative vertebral height (Mean ± SD)	14.89 ± 4.9	15.9 ± 4.59	13.17 ± 4.97
Preoperative compression ratio			
<20%	49(24.5)	40(31.8)	9(12.2)
≥20%, < 30%	48(24)	31(24.6)	17(22.9)
≥30%, < 40%	38(19)	29(23.0)	9(12.2)
>40%	65(32.5)	26(20.6)	39(52.7)
Sagittal index			
≤15°	131(65.5)	83(65.9)	48(64.9)
>15°	69(34.5)	43(34.1)	26(35.1)
Thoracolumbar kyphosis Cobb Angle			
<15°	60(30)	44(34.9)	16(21.6)
≥15°, < 25°	59(29.5)	39(31.0)	20(27.0)
≥25°, < 35°	42(21)	27(21.4)	15(20.3)
>35°	39(19.5)	16(12.7)	23(31.1)
Coronal Cobb Angle			
≤10°	150(75)	101(80.2)	49(66.2)
>10°	50(25)	25(19.8)	25(33.8)
Whether to carry out anti osteoporosis, *n* (%)			
No	111 (55.5)	58 (46)	53 (72)
Yes	89 (44.5)	68 (54)	21 (28)
Diffusion of bone cement, *n* (%)			
Bad	97 (48)	42 (33)	55 (74)
Good	103 (52)	84 (67)	19 (26)
Vertebral height recovery rate			
<60%	19(9.5)	5(4.0)	14(18.9)
≥60%, < 80%	46(23)	24(19.0)	22(29.7)
≥80%, < 100%	100(50)	70(55.6)	30(40.6)
>100%	35(17.5)	27(21.4)	8(10.8)
Whether bone cement fits the endplate, *n* (%)			
No	91 (45.5)	43 (34.1)	48 (64.9)
Yes	109 (54.5)	83 (65.9)	26 (35.1)

### LASSO Regression Analysis Results

**Fig. 1:**
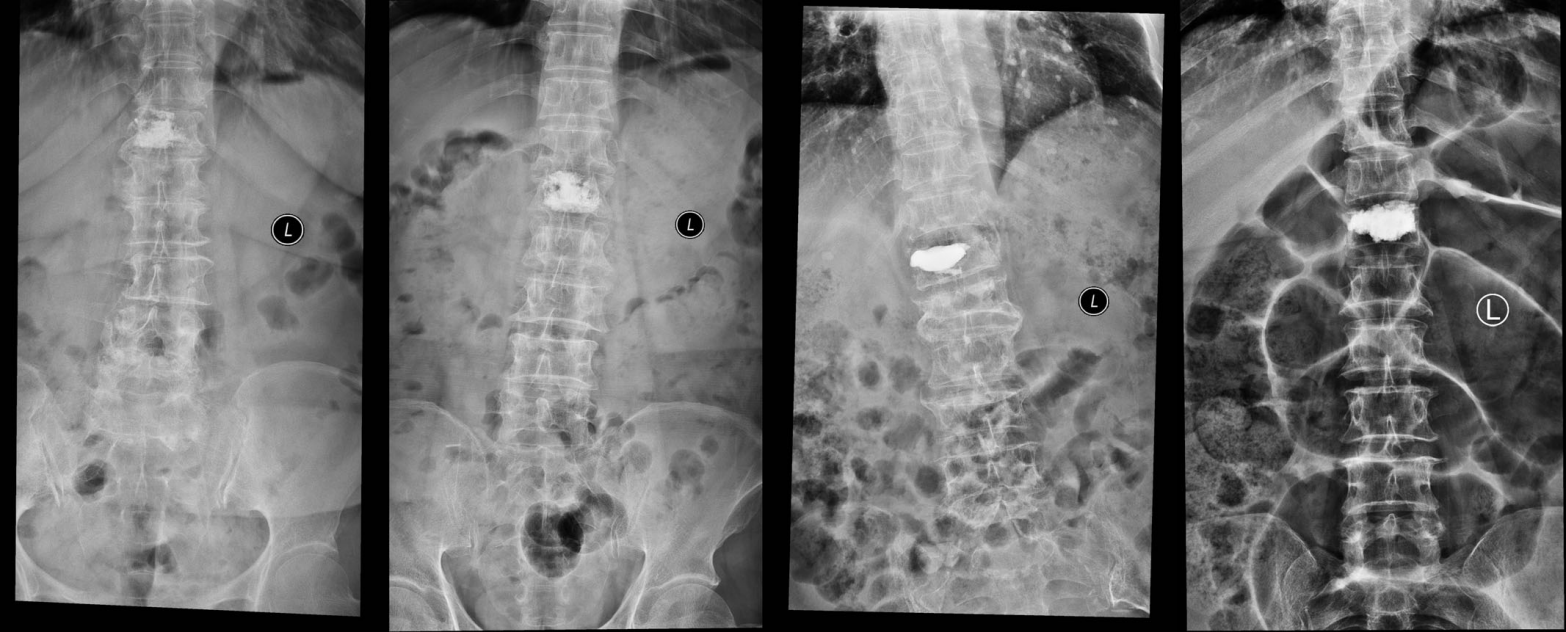
Observation of diffusion of bone cement and whether bone cement fits the endplate. (A) The vertebrae in which the bone cement located unilaterally (restricted by the midline) was defined as bad diffusion. (B) The vertebrae in which the bone cement diffused across the midline and evenly distributed inside was defined as good diffusion. (C) The vertebrae in which the bone cement had no contact with the upper or lower endplate. (D) The vertebrae in which the bone cement had some contacts with the upper or lower endplate

**Fig. 2:**
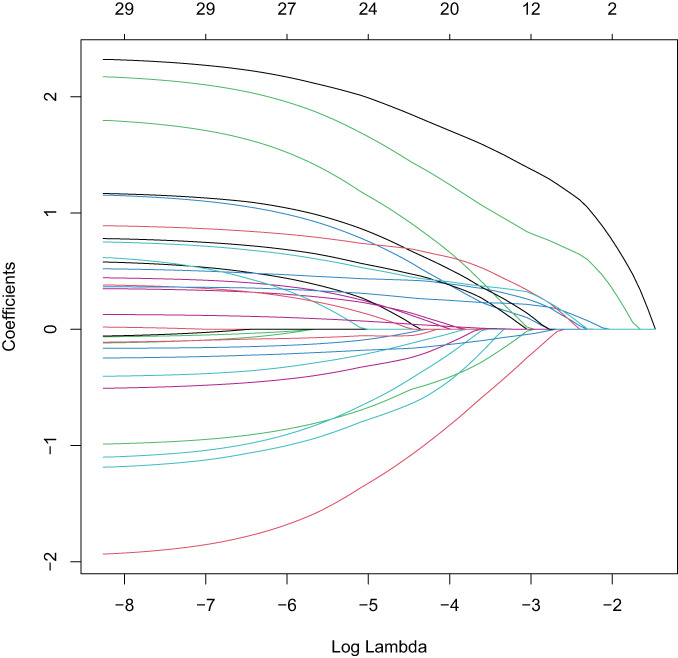
The variation characteristic of LASSO regression coefficient

**Fig. 3:**
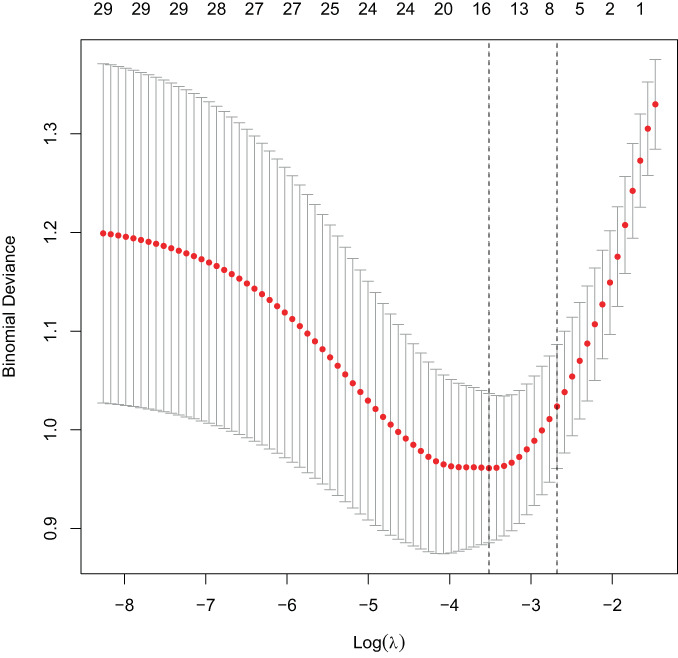
We selected the most suitable parameter lambda value for LASSO regression by cross‐validation, and the left dotted line represents the lambda value of the minimum objective parameter mean. In this case, the model achieves the best performance

LOSSA regression analysis was used to estimate the coefficient of each risk factor for NVCF. NVCF was used as a dependent variable and 29 predictive variables were incorporated into the LASSO regression model to select predictors related to NVCF, including time from injury to surgery, history of trauma, sex, age, BMI, parathyroid hormone, vitamin D, osteocalcin, β collagen special sequence, total type I collagen amino elongation peptide, red blood cell distribution width, fibrinogen, hemoglobin, activated partial thromboplastin time, homocysteine, alkaline phosphatase, serum Ca, serum creatinine, BMD, amount of bone cement injected, orthopaedic procedure, preoperative compression ratio, sagittal index, thoracolumbar kyphosis Cobb Angle, Coronal Cobb Angle, whether anti‐osteoporosis therapy was administered, diffusion of bone cement, vertebral height recovery rate, and whether the bone cement fit the endplate (Figures 2, 3). Fifteen variables were selected for preliminary screening, including time from injury to surgery, osteocalcin, total type I collagen amino elongation peptide, red blood cell distribution width, homocysteine, alkaline phosphatase, BMD, orthopaedic procedure, preoperative compression ratio, thoracolumbar kyphosis Cobb angle, coronal Cobb Angle, whether anti‐osteoporosis therapy was administered, diffusion of bone cement, vertebral height recovery rate, and whether the bone cement contacted the endplate. Multivariate logistic regression analysis showed that time from injury to surgery, osteocalcin, homocysteine, BMD, orthopaedic procedure, whether or not out anti‐osteoporosis therapy was administered and diffusion of bone cement (Figure 1) were independent predictors of NVCF (Table 2).

**Table 2:** Prediction factors for new vertebral compression fractures (NVCF)

Intercept and variable	Prediction model		
	β	Odds ratio [95% CI]	Pr(>|Z|)
Time from injury to surgery	2.3243	10.219000 [3.8700000 26.9850000]	<0.0001
Osteocalcin	−1.6297	0.038413 [0.0049862 0.2959300]	0.0018
Total type I collagen amino elongation peptide	0.6928	1.999400 [0.6584200 6.0713000]	0.2215
Red blood cell distribution width	−1.2266	0.086014 [0.0045954 1.6099000]	0.1007
Homocysteine	1.5351	4.641600 [1.4126000 15.2510000]	0.0114
Alkaline phosphatase	0.5539	3.027400 [0.3963100 23.1270000]	0.2856
BMD	1.0103	7.542800 [1.3206000 43.0800000]	0.0230
Orthopaedic procedures	−0.9639	0.381390 [0.1463000 0.9942600]	0.0486
Preoperative compression ratio	0.2373	1.607500 [0.6207500 4.1629000]	0.3282
Thoracolumbar kyphosis Cobb Angle	0.0037	1.007500 [0.4425100 2.2939000]	0.9858
Coronal Cobb Angle	0.9477	2.579800 [0.9029300 7.3707000]	0.0768
Whether to carry out anti osteoporosis	1.0234	2.782800 [1.0995000 7.0429000]	0.0308
Diffusion of bone cement	1.8057	6.084200 [2.2666000 16.3320000]	0.0003
Vertebral height recovery rate	−0.2958	0.743960 [0.3898500 1.4197000]	0.3697
Whether bone cement fits the endplate	0.5051	1.657200 [0.6739100 4.0749000]	0.2712

*Note*: β is the regression coefficient.

**Fig. 4:**
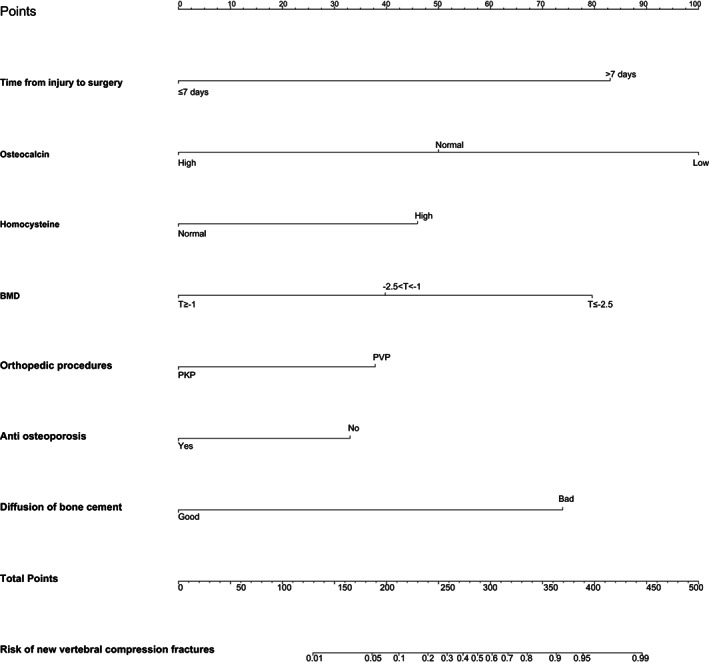
Each variable corresponds to the corresponding point on the corresponding variable axis of the nomogram, and the vertical line of the variable axis with this point corresponds to the upper scoring scale to obtain the variable score. The total score corresponds to the point on the NVCF risk axis below the nomogram, which is the patient's corresponding NVCF risk

**Fig. 5:**
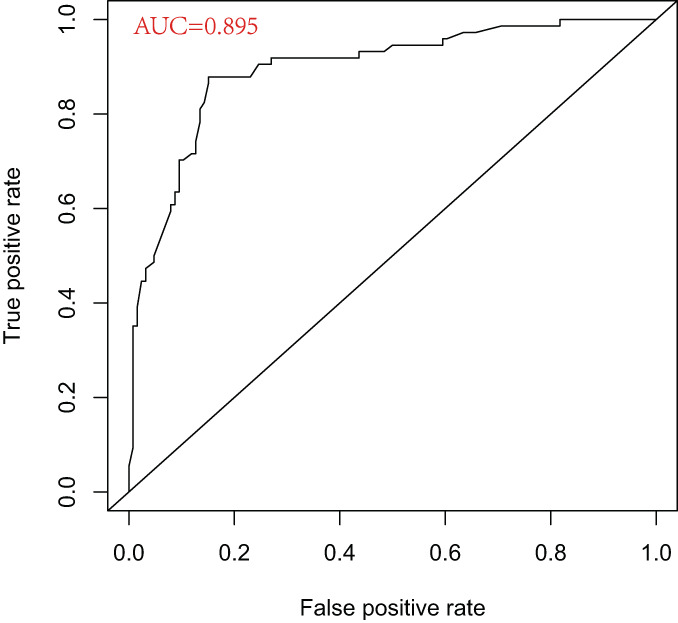
The ROC curve reflects the curve between sensitivity and specificity. The X axis is specific, also known as the false positive rate, the closer the X axis is to zero, the higher the accuracy; the larger the Y axis, the better the accuracy; the area under the curve (AUC) indicates the accuracy of prediction, and the higher the AUC value, the larger the area under the curve, the higher the accuracy of prediction

**Fig. 6:**
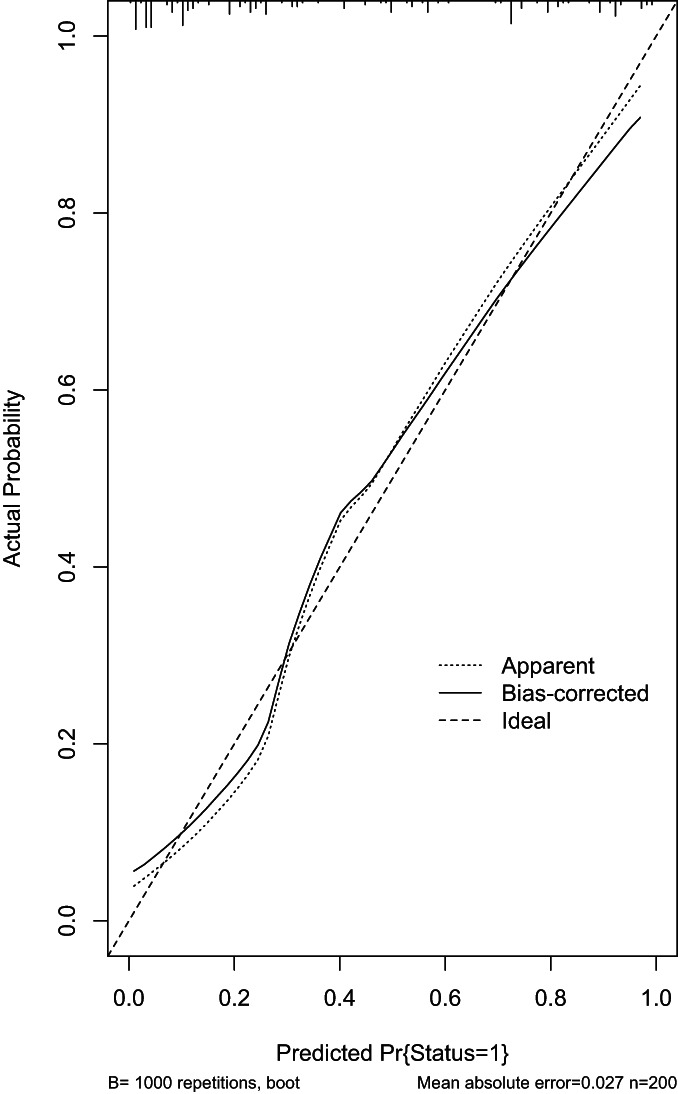
The fitting was repeated 1000 times, and the average absolute error was 0.027 (*n* = 200). The X axis represents the predicted NVCF risk; the Y axis represents the actual risk of NVCF; the diagonal dashed line represents the perfect prediction of the ideal model; and the solid line represents the performance of the line chart, which is closer to the diagonal dashed line to indicate a better prediction

**Fig. 7:**
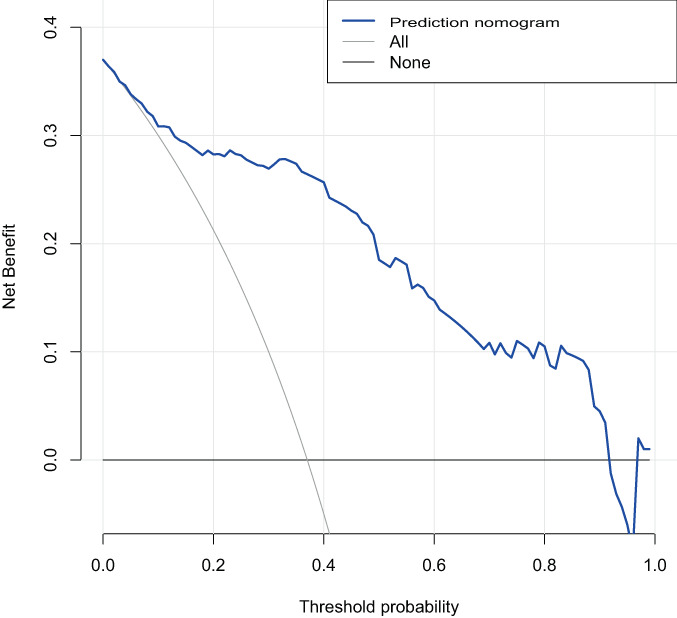
Decision curve analysis. The y‐axis represents net income. The blue curve represents the NVCF risk nomogram. The horizontal solid line represents the assumption that no patient has developed NVCF. The thin dotted line represents the hypothesis that NVCF occurs in all patients

### Development of an Individualized Prediction Model

A model with the above‐mentioned seven independent predictors was created for use with the nomogram (Figure 4). We plotted the calibration curve of the predictive model. The AUC of the predictive model was 0.895, demonstrating a high level of predictive accuracy (Figure 5). The apparent curve of the model fit well with the curve after bias correction, indicating that the predictive model had high discriminatory ability and good fit (Figure 6). After 1000 Bootstrap resampling verifications, it was confirmed that the concordance index was 0.866, which proved that the discriminatory ability of the model was excellent.

### Clinical Utility of the Nomogram for Predicting NVCF Risk

The results of line chart decision curve analysis for factors influencing NVCF showed that this line chart possessed the greatest clinical net benefit for predicting NVCF after bone cement injection if it is within the threshold range of 1% and 91%, as shown in Figure 7.

## Discussion

After our thorough study and exploration, we finally employed seven easily accessible factors to estimate the likelihood of postoperative NVCF, which would aid clinicians in implementing preoperative and postoperative management of patients in a scientific manner. To the best of our knowledge, our study is the first to combine patients' laboratory indicators with preoperative baseline characteristics and surgical treatment parameters for risk prediction into an easy‐to‐use nomogram, to evaluate the risk of NVCF in middle‐aged and elderly patients with vertebral compression fractures after cement injection.

### Effects of Different Surgical Procedure (PKP and PVP) on NVCF

Both PKP and PVP are safe and efficient minimally invasive surgical techniques for the treatment of vertebral compression fractures. Polymethyl methacrylate cement is injected into the fractured vertebral body to achieve mechanical stabilization of the fractured vertebral body, improve the strength of the vertebral body, and relieve pain.^14^ However, experts are divided about the procedure with the maximal clinical benefit, especially with respect to the degree of amelioration of postoperative pain symptoms and the probability of NVCF. Griffoni et al.^15^ investigated the efficacy and safety of PKP and PVP for vertebral compression fracture using long‐term follow‐up; they found that both methods resulted in good recovery of vertebral height and improvement of the kyphosis angle, but the risk of adjacent segmental fractures was significantly higher in the PVP group. A network analysis^16^ showed that PKP was the best treatment to enhance the quality of life and lower the risk of vertebral re‐fracture compared to PVP and conservative treatment, and the risk of adjacent vertebral fracture was substantially lower with PKP than that with PVP. Several studies have linked poor spinal sagittal alignment with a higher risk of vertebral compression fracture in individuals with osteoporosis; moreover, the worse the overall sagittal position, the poorer the quality of life.^17^ Cao et al.^18^ found that PKP can significantly improve global sagittal plane imbalance caused by vertebral fractures, especially in vertebral fractures involving the thoracolumbar segment, and correct pelvic posterior rotation occurring during sagittal compensatory balance within a short time, which is crucial for realizing the balance between spinal and pelvic sagittal parameters and maintaining the axis of gravity in the natural position as far as possible. Therefore, these studies provide robust evidence to support screening of risk factors and model development. Additionally, we believe that PKP may be superior to PVP with respect to the lower incidence of postoperative secondary vertebral compression fractures and better quality of life, based on our analysis and previous studies.

### Preventive Effects of Osteocalcin on NVCF

Osteocalcin, a bone biochemical marker secreted by osteoblasts, is the most abundant non‐collagenous protein in bone, which is essential for the synthesis and turnover of bone matrix.^19^, ^20^ The plasma level of osteocalcin is thought to reflect the degree of bone formation.^21^ Osteocalcin, in conjunction with other bone biochemical markers, can be used to quantitatively monitor the level of bone metabolism. Utilizing these quantitative changes for risk prediction may bolster timely and comprehensive evaluation during the bone healing phase and predict the risk of complications and the degree of damage to fracture healing.^22^ Poundarik et al.^23^ found that dilatational bands formed by the osteocalcin–osteopontin interaction are vital for energy dissipation and maintaining the fracture toughness of bone. Moreover, empirical evidence supports the significant role of osteocalcin in bone fracture resistance. Nikel et al.^24^ were the first to prove the importance of osteocalcin and osteopontin in creep and fatigue of bone, and demonstrated that their deficiency seriously compromised the plasticity of the bone matrix and its ability to withstand periodic load. Therefore, osteocalcin plays a key role in improving the toughness and anti‐fracture properties of bone, which has a far‐reaching impact on the prevention of NVCF after bone cement injection.

### Effects of Excessive Homocysteine on NVCF

Numerous experimental studies have shown that homocysteine is an independent risk factor for vertebral compression fractures in the elderly; elevated homocysteine levels increase the risk of fractures in humans.^25^, ^26^, ^27^ Homocysteine can reduce bone vascular flow, which can destroy the cross‐linking of collagen molecules and affect the normal calcification process.^28^, ^29^ Additionally, excessive homocysteine can induce apoptosis of osteoblasts, osteocytes, and human bone marrow stromal cells by enhancing the differentiation of osteoclasts, resulting in damage to the mechanical strength of bone.^28^, ^29^ This exerts adverse effects on the healing of fracture sites, balance of bone metabolism, and the recovery of bone mechanical properties after bone cement injection. Substantial evidence indicates that the decline in homocysteine levels can reduce the risk of cardiovascular and cerebrovascular diseases such as stroke, delay the decline of coordination function, increase bone strength in the elderly, and reduce the risk of fall secondary to skeletal muscle weakness, thus reducing the risk of vertebral compression fracture in the elderly.^30^, ^31^, ^32^ According to a Japanese study, homocysteine was an independent risk factor for moderate and severe vertebral compression fractures; the risk of subsequent vertebral fractures and other osteoporotic fractures was considerably higher in patients with severe fractures than that in patients with moderate fractures.^33^, ^34^ According to the above‐mentioned study, homocysteine elevation may have an adverse effect on the patients' ability to recover after bone cement injection and increase the risk of NVCF.

### Effects of Surgical Treatment and Anti‐Osteoporosis Treatment on NVCF

Osteoporosis can lead to the destruction of the microstructure of the human bone and decrease in bone strength.^35^ Patients with osteoporosis are susceptible to compression fracture and other fragility fractures, while patients with vertebral compression fractures may experience serious physical limitations, such as back pain and dysfunction.^36^ If not treated as soon as possible, the height of the vertebral body may collapse further with the increase in the fracture age and range of motion of the fracture site, and the repeated compressive load at the fracture site may lead to bone loss near the endplate, which may increase the risk of NVCF.^37^ Clinical studies with long‐term follow‐up have shown that regular administration of anti‐osteoporotic drugs, such as teriparatide or zoledronic acid, to post‐PKP patients with osteoporotic vertebral compression fractures can significantly reduce the incidence of NVCF and back pain, increase BMD, effectively restore postoperative function, and improve patients' quality of life, consistent with the present study.^38^, ^39^ Therefore, we believe that early treatment of fractures and targeted anti‐osteoporosis treatment play a key role in the prevention of NVCF.

Several studies have focused on the degree of diffusion of bone cement, which exerts a significant impact on biomechanical aspects, such as the stability and mechanical properties of the fracture site. Some studies have shown that the asymmetric distribution of the vertebral body and excessive filling of cement volume lead to the transfer and switching of unilateral load, resulting in NVCF.^40^, ^41^ The height and strength of the vertebral body recovered well, and the risk of vertebral recompression decreased significantly when the bone cement was in contact with the upper and lower endplates.^40^, ^41^ At the same time, some experts believe that when the cement distribution is wide enough (i.e., the area between the midline and contralateral pedicle), unilateral PKP can significantly restore the height of the vertebral body and provide a good therapeutic effect.^15^ Therefore, good cement diffusion has a positive impact on fracture site stability, mechanical properties, and stress distribution, effectively preventing NVCF in the vertebral body.

### Clinical Significance of the Study

Although previous studies have conducted in‐depth and extensive investigations into the effects of osteocalcin and homocysteine on vertebral compression fractures and bone metabolic balance in middle‐aged and elderly people, no study has investigated their association with the incidence of NVCF after bone cement injection. In the current study, osteocalcin and homocysteine were combined with preoperative baseline characteristics and surgical treatment factors to comprehensively study the risk of NVCF. Notably, no study has comprehensively investigated the risk of NVCF by combining osteocalcin and homocysteine with preoperative baseline characteristics and surgical treatment factors as risk factors. By incorporating all relevant risk factors, our study provides a comprehensive, accurate, and easy‐to‐implement nomogram to calculate the probability of NVCF in patients after bone cement injection surgery, which may have profound implications for postoperative prevention, treatment, and targeted follow‐up, and also serve as a roadmap for future studies.

### Strengths and Limitations

Nomograms are a scientific tool with high accuracy and provides us with easy‐to‐understand prognostic models. Our strength is to take advantage of nomograms and comprehensively combine patients' laboratory indicators with preoperative baseline characteristics and surgical treatment parameters to produce a risk prediction model which is conducive to clinical decision‐making with high credibility and easy operation.

This study is beset by several limitations. First, although our nomogram was robust and underwent thorough internal verification using Bootstrap sampling, the clinical data were acquired from our hospital's orthopaedic department. Hence, the lack of comparison with data from other centres, and lack of external verification, necessitate external evaluation in other middle‐aged and elderly populations with vertebral fractures from more countries and regions. Although we comprehensively collected the clinical data from our sample, another limitation is the small sample size, and selection bias resulting from the retrospective study design. Therefore, it is necessary to perform collaborative multi‐center studies to further augment our data and prospective research to verify the accuracy of this nomogram. The current analysis investigated the influence of osteocalcin and homocysteine on NVCF after bone cement injection only from the macrostructural perspective. Further cellular and molecular studies are required to elucidate the biological mechanisms associated with osteocalcin and homocysteine and fracture susceptibility or factors leading to new fractures after surgery, to verify the model's correctness from a microscopic perspective.

### Conclusion

Time from injury to surgery more than 7 days, osteocalcin levels below normal, homocysteine levels above the normal range, presence of osteoporosis, mode of operation, that is, percutaneous vertebroplasty, lack of postoperative anti‐osteoporosis treatment, and poor diffusion of bone cement are independent risk factors of NVCF in middle‐aged and elderly patients with vertebral compression fracture after bone cement injection. The nomogram based on these seven factors can objectively and accurately prognosticate the likelihood of NVCF in middle‐aged and elderly patients with vertebral compression fracture following bone cement injection and assist clinicians in timely determination of the risk of clinical events and development of individualized treatment plans. Future large‐scale studies are warranted to determine the predictive efficiency of the model and explore whether targeted postoperative patient management using the model's predictions can reduce the probability of NVCF.

## Author Contributions

All authors had full access to the data in the study and take responsibility for the integrity of the data and the accuracy of the data analysis. Conceptualization, Wenxin Gao and Yanke Hao; Methodology, Wenxin Gao and Yanke Hao; Investigation, Xiaoying Wang and Guoyan Liu; Formal Analysis, Wenxin Gao and Kaiying Cui; Resources, Kaiying Cui and Jinxing Guo; Writing—Original Draft, Wenxin Gao and Yungang Chen; Writing—Review & Editing, Yanke Hao; Visualization, jianhuzheng; Supervision, Xiaoying Wang; Funding Acquisition, Yanke Hao.

## Conflict Of Interest

We declare that the research was conducted in the absence of any commercial or financial relationships that could be construed as a potential conflict of interest.

## Ethics Statement

The study protocol was approved by the Affiliated Hospital of Shandong University of Traditional Chinese Medicine's institutional research ethics committee.

## References

[1] Burge R , Dawson‐Hughes B , Solomon DH , Wong JB , King A , Tosteson A . Incidence and economic burden of osteoporosis‐related fractures in the United States, 2005‐2025. Journal of Bone and Mineral Research: The Official Journal of the American Society for Bone and Mineral Research. 2007;22(3):465–75. 17144789 10.1359/jbmr.061113

[2] Johnell O , Kanis JA . An estimate of the worldwide prevalence and disability associated with osteoporotic fractures. Osteoporos Int. 2006;17(12):1726–33. 16983459 10.1007/s00198-006-0172-4

[3] Wardlaw D , Cummings SR , Van Meirhaeghe J , Bastian L , Tillman JB , Ranstam J , et al. Efficacy and safety of balloon kyphoplasty compared with non‐surgical care for vertebral compression fracture (FREE): a randomised controlled trial. Lancet. 2009;373(9668):1016–24. 19246088 10.1016/S0140-6736(09)60010-6

[4] Musbahi O , Ali AM , Hassany H , Mobasheri R . Vertebral compression fractures. Br J Hosp Med (Lond). 2018;79(1):36–40. 29315051 10.12968/hmed.2018.79.1.36

[5] Feng L , Feng C , Chen J , Wu Y , Shen J‐M . The risk factors of vertebral refracture after kyphoplasty in patients with osteoporotic vertebral compression fractures: a study protocol for a prospective cohort study. BMC Musculoskelet Disord. 2018;19(1):195. 29961425 10.1186/s12891-018-2123-6 PMC6027566

[6] Chen Z , Song C , Lin H , Sun J , Liu W . Does prophylactic vertebral augmentation reduce the refracture rate in osteoporotic vertebral fracture patients: a meta‐analysis. Eur Spine J. 2021;30(9):2691–7. 34132903 10.1007/s00586-021-06899-w

[7] Li Q , Long X , Wang Y , Fang X , Guo D , Lv J , et al. Development and validation of a nomogram for predicting the probability of new vertebral compression fractures after vertebral augmentation of osteoporotic vertebral compression fractures. BMC Musculoskelet Disord. 2021;22(1):957. 34784910 10.1186/s12891-021-04845-x PMC8597210

[8] Park J‐S , Park Y‐S . Survival analysis and risk factors of new vertebral fracture after vertebroplasty for osteoporotic vertebral compression fracture. Spine J. 2021;21(8):1355–61. 33971326 10.1016/j.spinee.2021.04.022

[9] Wang H , Chen X , Zhao J , Kang M , Dong R , Wang K , et al. Predictive nomogram for midterm to Long‐term prognosis in patients with papillary renal cell carcinoma based on data from the surveillance, epidemiology, and end results (SEER) program. Med Sci Monit. 2020;26:e921859. 32570266 10.12659/MSM.921859 PMC7331481

[10] Collins GS , Reitsma JB , Altman DG , Moons KGM . Transparent reporting of a multivariable prediction model for individual prognosis or diagnosis (TRIPOD): the TRIPOD statement. BMJ. 2015;350:g7594. 25569120 10.1136/bmj.g7594

[11] Hu X , Shen F , Zhao Z , Qu X , Ye J . An individualized gait pattern prediction model based on the least absolute shrinkage and selection operator regression. J Biomech. 2020;112:110052. 33039924 10.1016/j.jbiomech.2020.110052

[12] Wang H , Zhang L , Liu Z , Wang X , Geng S , Li J , et al. Predicting medication nonadherence risk in a Chinese inflammatory rheumatic disease population: development and assessment of a new predictive nomogram. Patient Prefer Adherence. 2018;12:1757–65. 30237698 10.2147/PPA.S159293 PMC6136915

[13] Li W , Wang H , Dong S , Tang Z‐R , Chen L , Cai X , et al. Establishment and validation of a nomogram and web calculator for the risk of new vertebral compression fractures and cement leakage after percutaneous vertebroplasty in patients with osteoporotic vertebral compression fractures. Eur Spine J. 2022;31(5):1108–21. 34822018 10.1007/s00586-021-07064-z

[14] Long Y , Yi W , Yang D . Advances in vertebral augmentation Systems for Osteoporotic Vertebral Compression Fractures. Pain Res Manag. 2020;2020:3947368–9. 33376566 10.1155/2020/3947368 PMC7738798

[15] Griffoni C , Lukassen JNM , Babbi L , Girolami M , Lamartina C , Cecchinato R , et al. Percutaneous vertebroplasty and balloon kyphoplasty in the treatment of osteoporotic vertebral fractures: a prospective randomized comparison. Eur Spine J. 2020;29(7):1614–20. 32361843 10.1007/s00586-020-06434-3

[16] Zhu RS , Kan SL , Ning GZ , Chen LX , Cao ZG , Jiang ZH , Zhang XL , Hu W Which is the best treatment of osteoporotic vertebral compression fractures: balloon kyphoplasty, percutaneous vertebroplasty, or non‐surgical treatment? A Bayesian network meta‐analysis Osteoporos Int 2019;30(2):287–98. 10.1007/s00198-018-4804-2 30635698

[17] Chau LTC , Hu Z , Ko KSY , Man GCW , Yeung KH , Law YY , et al. Global sagittal alignment of the spine, pelvis, lower limb after vertebral compression fracture and its effect on quality of life. BMC Musculoskelet Disord. 2021;22(1):476. 34030686 10.1186/s12891-021-04311-8 PMC8146251

[18] Cao Z , Wang G , Hui W , Liu B , Liu Z , Sun J . Percutaneous kyphoplasty for osteoporotic vertebral compression fractures improves spino‐pelvic alignment and global sagittal balance maximally in the thoracolumbar region. PLoS One. 2020;15(1):e0228341. 31999783 10.1371/journal.pone.0228341 PMC6992186

[19] Fusaro M , Cianciolo G , Brandi ML , Ferrari S , Nickolas TL , Tripepi G , et al. Vitamin K and osteoporosis. Nutrients. 2020;12:12. 10.3390/nu12123625 PMC7760385 33255760

[20] Pan C , Liu X , Li T , Wang G , Sun J . Kinetic of bone turnover markers after osteoporotic vertebral compression fractures in postmenopausal female. J Orthop Surg Res. 2018;13(1):314. 30526635 10.1186/s13018-018-1025-5 PMC6286497

[21] Wei F‐F , Trenson S , Verhamme P , Vermeer C , Staessen JA . Vitamin K‐dependent matrix Gla protein as multifaceted protector of vascular and tissue integrity. Hypertension. 2019;73(6):1160–9. 31006332 10.1161/HYPERTENSIONAHA.119.12412 PMC6510326

[22] Sousa CP , Dias IR , Lopez‐Peña M , Camassa JA , Lourenço PJ , Judas FM , et al. Bone turnover markers for early detection of fracture healing disturbances: a review of the scientific literature. An Acad Bras Cienc. 2015;87(2):1049–61. 25993365 10.1590/0001-3765201520150008

[23] Poundarik AA , Diab T , Sroga GE , Ural A , Boskey AL , Gundberg CM , et al. Dilatational band formation in bone. Proc Natl Acad Sci U S A. 2012;109(47):19178–83. 23129653 10.1073/pnas.1201513109 PMC3511118

[24] Nikel O , Poundarik AA , Bailey S , Vashishth D . Structural role of osteocalcin and osteopontin in energy dissipation in bone. J Biomech. 2018;80:45–52. 30205977 10.1016/j.jbiomech.2018.08.014 PMC6188842

[25] Yang J , Hu X , Zhang Q , Cao H , Wang J , Liu B . Homocysteine level and risk of fracture: a meta‐analysis and systematic review. Bone. 2012;51(3):376–82. 22749888 10.1016/j.bone.2012.05.024

[26] Périer MA , Gineyts E , Munoz F , Sornay‐Rendu E , Delmas PD . Homocysteine and fracture risk in postmenopausal women: the OFELY study. Osteoporos Int. 2007;18(10):1329–36. 17549579 10.1007/s00198-007-0393-1

[27] Leboff MS , Narweker R , LaCroix A , Wu L , Jackson R , Lee J , et al. Homocysteine levels and risk of hip fracture in postmenopausal women. J Clin Endocrinol Metab. 2009;94(4):1207–13. 19174498 10.1210/jc.2008-1777 PMC2682463

[28] Wang P , Liu L , Lei S‐F . Causal effects of homocysteine levels on the changes of bone mineral density and risk for bone fracture: a two‐sample mendelian randomization study. Clin Nutr. 2021;40(4):1588–95. 33744603 10.1016/j.clnu.2021.02.045

[29] Saito M , Marumo K . The effects of homocysteine on the skeleton. Curr Osteoporos Rep. 2018;16(5):554–60. 30116976 10.1007/s11914-018-0469-1

[30] Huang X , Li Y , Li P , Li J , Bao H , Zhang Y , et al. Association between percent decline in serum total homocysteine and risk of first stroke. Neurology. 2017;89(20):2101–7. 29030456 10.1212/WNL.0000000000004648

[31] He T , Jin X , Koh YS , Zhang Q , Zhang C , Liu F . The association of homocysteine, folate, vitamin B12, and vitamin B6 with fracture incidence in older adults: a systematic review and meta‐analysis. Ann Transl Med. 2021;9(14):1143. 34430584 10.21037/atm-21-2514 PMC8350623

[32] Zhu Y , Shen J , Cheng Q , Fan Y , Lin W . Plasma homocysteine level is a risk factor for osteoporotic fractures in elderly patients. Clin Interv Aging. 2016;11:1117–21. 27574411 10.2147/CIA.S107868 PMC4993272

[33] Kuroda T , Tanaka S , Saito M , Shiraki Y , Shiraki M . Plasma level of homocysteine associated with severe vertebral fracture in postmenopausal women. Calcif Tissue Int. 2013;93(3):269–75. 23793599 10.1007/s00223-013-9754-2

[34] Roux C , Fechtenbaum J , Kolta S , Briot K , Girard M . Mild prevalent and incident vertebral fractures are risk factors for new fractures. Osteoporos Int. 2007;18(12):1617–24. 17611706 10.1007/s00198-007-0413-1

[35] Johnston CB , Dagar M . Osteoporosis in older adults. Med Clin North Am. 2020;104(5):873–84. 32773051 10.1016/j.mcna.2020.06.004

[36] Hoyt D , Urits I , Orhurhu V , Orhurhu MS , Callan J , Powell J , et al. Current concepts in the Management of Vertebral Compression Fractures. Curr Pain Headache Rep. 2020;24(5):16. 32198571 10.1007/s11916-020-00849-9

[37] Yang C‐C , Chien J‐T , Tsai T‐Y , Yeh K‐T , Lee R‐P , Wu W‐T . Earlier Vertebroplasty for osteoporotic thoracolumbar compression fracture may minimize the subsequent development of adjacent fractures: a retrospective study. Pain Physician. 2018;21(5):E483–E91. 30282396

[38] Lu K , Yin Y , Li C , Jin Y , Shan HQ . Efficacy of annual zoledronic acid in initial percutaneous kyphoplasty patients with osteoporotic vertebral compression fractures: a 3‐year follow‐up study. Osteoporos Int. 2021;32(7):1429–39. 33462653 10.1007/s00198-020-05816-z

[39] Kong M , Zhou C , Zhu K , Zhang Y , Song M , Zhang H , et al. 12‐month Teriparatide treatment reduces new vertebral compression fractures incidence and Back pain and improves quality of life after percutaneous Kyphoplasty In osteoporotic women. Clin Interv Aging. 2019;14:1693–703. 31631990 10.2147/CIA.S224663 PMC6778479

[40] Liebschner MA , Rosenberg WS , Keaveny TM . Effects of bone cement volume and distribution on vertebral stiffness after vertebroplasty. Spine (Phila Pa 1976). 2001;26(14):1547–54. 11462084 10.1097/00007632-200107150-00009

[41] Peng J , Qin J , Huang T , Luo X , Zhong W , Quan Z . Clinical outcomes of fracture Haemorrhage aspiration for percutaneous Vertebroplasty in treating osteoporotic vertebral compression fractures. J Pain Res. 2021;14:3951–9. 35002314 10.2147/JPR.S345760 PMC8725857

